# Innovative nomogram for cervical cancer prediction: integrating high-risk HPV infection, *p53* genotype, and blood routine parameters

**DOI:** 10.3389/fonc.2025.1541928

**Published:** 2025-05-20

**Authors:** Cheng Sun, Jun Zhang, Lili Pan, Shuang Yao, Fenghua Zhang, Linjuan Ji, Miaomei Yu, Guanghua Luo, Xiping Jiang

**Affiliations:** ^1^ Department of Gynecology, The First People’s Hospital of Changzhou and the Third Affiliated Hospital of Soochow University, Changzhou, China; ^2^ Clinical Medical Research Center, The First People’s Hospital of Changzhou and the Third Affiliated Hospital of Soochow University, Changzhou, China; ^3^ Changzhou Medical Center, Nanjing Medical University, Changzhou, China

**Keywords:** cervical cancer, blood routine parameters, p53, high-risk HPV, nomogram

## Abstract

**Background:**

Human papillomavirus (HPV) infection, especially high-risk types like HPV16 and HPV18, is a primary cause of cervical cancer. The *p53* gene influences cellular response to DNA damage and has a functional polymorphism (rs1042522, p.Arg72Pro) that affects susceptibility to degradation by HPV E6 protein. This study aims to analyze the relationship among *p53* genotypes, high-risk HPV infection, and hematological parameters in cervical cancer development and to develop a predictive model.

**Methods:**

This retrospective cross-sectional study collected cervical cancer specimens and brush samples from patients at the First People’s Hospital of Changzhou between January 2020 and August 2024. HPV types and *p53* genotyping were performed using PCR. Inflammatory markers like neutrophil-to-lymphocyte ratio (NLR), systemic immune-inflammation index (SII), and platelet-to-lymphocyte ratio (PLR) were calculated. Statistical analyses including logistic regression and LASSO were used to construct a predictive model.

**Results:**

The study included 147 female patients with cervical cancer and controls. HPV16 and HPV18 had high infection rates. In the log-additive model, each additional *p53* C allele reduced the risk by 48% (OR = 0.52, 95% CI: 0.27-0.98, *P* = 0.038). Significant interactions were found between *p53* genotypes and HPV18 infection on cervical cancer risk (*P* = 0.026). Cervical cancer patients showed reduced red blood cell count and hemoglobin. The predictive model, including *p53* genotype, HPV16, HPV18, and hematological parameters, had an AUC of 0.920 (95% CI: 0.875–0.965).

**Conclusion:**

The study identified significant differences in *p53* genotypes, HPV infection, and hematological parameters between cervical cancer patients and controls. The predictive model demonstrated high discriminatory ability for cervical cancer risk assessment. The interaction between HPV18 and *p53* genotypes suggests a potential protective effect of the *p53* C allele. Larger studies are needed to validate these findings.

## 1 Introduction

Cervical cancer is one of the most common malignant tumors among women worldwide, especially in developing countries, where the incidence and mortality rates remain high (1). Human papillomavirus (HPV) infection is considered the main cause of cervical cancer, particularly high-risk HPV types (such as HPV16 and HPV18), which are closely associated with the development of the disease (2–5). However, not all women infected with high-risk HPV will develop cervical cancer, suggesting that individual genetic susceptibility may play an important role in this process (6, 7). The *p53* gene, a critical tumor suppressor, may influence the cellular response to DNA damage when mutated or polymorphic. Studies have shown that the *p53* gene has a functional polymorphism (rs1042522, p.Arg72Pro), where the p.72Arg variant of *p53* is more susceptible to degradation mediated by the HPV E6 protein compared to the p.72Pro variant. Therefore, individuals carrying the p.72Arg variant of *p53* have a significantly higher risk of developing cervical cancer (8, 9). However, no consistent conclusions have been reached across different ethnic populations (10, 11). This study will further analyze the relationship between *p53* genotypes and the risk of cervical cancer.

In recent years, researchers have gradually recognized the potential value of hematological indicators in the early diagnosis and prognosis of tumors. Inflammatory markers such as the neutrophil to lymphocyte ratio (NLR), the systemic immune-inflammation index (SII), and the platelet to lymphocyte ratio (PLR) are considered to be closely related to the occurrence, progression, and prognosis of tumors (12–14). Thus, the integration of HPV infection status, *p53* genotype, and routine blood parameters may offer a new approach for the preliminary screening and risk assessment of cervical cancer. This study aims to investigate the interrelationships between high-risk HPV infection, *p53* genotypes, and routine blood parameters in the development of cervical cancer, identify factors associated with its onset, and develop an effective nomogram predictive model to support clinical decision-making.

## 2 Materials and methods

### 2.1 Study design and participants

This study is a retrospective cross-sectional study that collected cervical cancer specimens from patients who underwent surgical treatment at the First People’s Hospital of Changzhou between January 2020 and August 2024 (cervical cancer group, 55 cases). Additionally, cervical brush samples were collected from women with normal or benign lesions (cervicitis or cervical intraepithelial neoplasia grade I) diagnosed by pathology following cervical biopsy at the same Hospital during the same period (control group, 92 cases).

Inclusion criteria: 1) Age between 18–75 years; 2) No use of medications that could affect study outcomes within the past two weeks; 3) Availability of complete clinical data.

Exclusion criteria: 1) History of cervical surgery; 2) History of pelvic radiotherapy; 3) History of chemotherapy; 4) Presence of severe heart, liver, or kidney disease; 5) Presence of autoimmune diseases; 6) Co-existing with other malignancies.

### 2.2 PCR analysis system components and reagents

The fully automated medical PCR analysis system SLAN-96S (Shanghai Hongshi Medical Technology Co., Ltd., China) was used. PCR reaction system included: 10× buffer, 50 mM MgCl_2_, IMMOLASE™ DNA polymerase (Midian Biotechnology Inc., USA), and dNTPs (Takara, Japan). The quantitative PCR reaction system included: 10× buffer, 25 mM MgCl_2_, Taq DNA polymerase, dNTPs (Shanghai Bocai Biotechnology Co., Ltd., China), and the SYSMEX XN-9000 fully automated blood analyzer (Sysmex Corporation, Japan).

### 2.3 Typing method for high-risk HPV and *p5*3 and *RB1*

The method established by Zhang Jun et al. (15), was used to detect 16 types of high-risk HPV and related tumor suppressor genes, *p53* and *RB1*. This method is based on high-throughput two-dimensional PCR technology (2D-PCR) (16). Briefly, specific primers are designed according to the DNA sequences of 16 different types of high-risk HPV, as well as the *p53* and *RB1* genes. The upstream primers for the different types of high-risk HPV, *p53*, and *RB1* are labeled with corresponding tags. After the PCR reaction is completed, a melting curve analysis is performed. In three fluorescence detection channels, the probes and the complementary sequences of corresponding tags bind together and dissociate as the temperature increases, resulting in clearly distinguishable melting valleys, which allows for accurate determination of the genotypes.

### 2.4 Blood routine parameters

A fully automated blood analyzer was utilized to analyze the routine indicators of peripheral venous blood from the subjects included in this study. These indicators included white blood cell count (WBC), red blood cell count (RBC), hemoglobin (HGB), platelet count (PLT), neutrophil count (NEUT), neutrophil percentage (NEUT%), eosinophil count (EO), eosinophil percentage (EO%), basophil count (BASO), basophil percentage (BASO%), lymphocyte count (LY), lymphocyte percentage (LY%), monocyte count (MONO), monocyte percentage (MONO%), hematocrit (HCT), red cell distribution width-coefficient of variation (RDW-CV), mean corpuscular hemoglobin (MCH), and mean corpuscular hemoglobin concentration (MCHC).

The inflammatory indicators calculated based on the above complete blood count parameters included the systemic inflammation response index (SIRI), NLR, SII, PLR, lymphocyte to monocyte ratio (LMR), and neutrophil to platelet ratio (NPR). The formulas for calculation were as follows:

- SIRI = NEUT × MONO/LY
- NLR = NEUT/LY
- SII = PLT × NEUT/LY
- PLR = PLT/LY
- LMR = LY/MONO
- NPR = NEUT/PLT

### 2.5 Statistical analysis

The SNPStats online analysis tool (https://www.snpstats.net/start.htm) was employed to examine the allele and genotype frequencies of the *p53* gene, a cervical cancer susceptibility gene, and its interaction with high-risk HPV infection. Continuous variables that followed a normal distribution were presented as means with standard deviations (SD), while non-normally distributed continuous variables were described using medians and interquartile ranges (IQR). Categorical variables were summarized using counts and percentages (%).

The process for constructing the optimal clinical predictive model for cervical cancer diagnosis involved several steps. First, a univariate logistic regression analysis was performed on all variables to identify those with a *P*-value below 0.20, which were selected as candidate variables for further modeling. Second, the least absolute shrinkage and selection operator (LASSO) regression was applied to refine these candidate variables, using 10-fold cross-validation to determine the optimal regularization parameter (λ). Next, the variables selected through LASSO regression were incorporated into a multivariate logistic regression analysis, with the final variables being selected via a backward stepwise method (BACKWARD) to construct the nomogram prediction model.

Subsequently, the model’s discriminative ability was assessed using a receiver operating characteristic (ROC) curve, calibration was evaluated through a calibration curve, and the clinical applicability of the model was analyzed using decision curve analysis (DCA). To further assess and validate the model’s performance, 1000 bootstrap resampling iterations were conducted.

### 2.6 Ethics statement

This study was approved by the Ethics Committee of the First People’s Hospital of Changzhou on December 17, 2023 (Approval No (2023):195). The Ethics Committee granted a waiver of written informed consent due to the retrospective nature of the study and the anonymization of all samples prior to analysis.

## 3 Results

### 3.1 Study population characteristics and HPV infection rates

As shown in **Supplementary Table S1**, a total of 147 female patients were included in this study. The mean age was 48.76 ± 11.26 years (range: 20–74 years). The mean age of the cervical cancer group was significantly higher than that of the control group (*P =* 0.023). Among the total participants, HPV16 and HPV18 had relatively high infection rates, accounting for 22% and 12%, respectively. Significant differences in high-risk HPV infection rates between the cervical cancer and control groups were observed for HPV16 (*P <* 0.001), HPV18 (*P =* 0.027), and HPV58 (*P =* 0.032).

All 147 patients in this study had the A/A genotype for the *RB1* gene, and thus, this gene was excluded from further analysis. The allele and genotype frequencies of the *p53* gene are shown in **Supplementary Table S2**. The Hardy-Weinberg equilibrium exact test results indicated *P >* 0.05 (**Supplementary Table S3**), suggesting that the study population was genetically stable and representative, providing a solid foundation for subsequent association analyses and interaction studies with high-risk HPV.

### 3.2 Adjusted association analysis of *p53* gene variants with cervical cancer risk

After adjusting for age and multiple high-risk HPV types, including HPV16, HPV18, HPV31, HPV33, HPV39, HPV45, HPV51, HPV52, HPV56, HPV58, HPV59, HPV66, HPV68, and HPV82, the association analysis between the *p53* gene and cervical cancer is presented in **Table 1**. In the codominant model, using the G/G genotype as the reference group, the C/C genotype showed a 76% reduction in risk (OR = 0.24, 95% CI: 0.06-0.95), although this was not statistically significant (*P >* 0.05). In the log-additive model, each additional C allele was associated with a 48% reduction in cervical cancer risk, which was statistically significant (OR = 0.52, 95% CI: 0.27-0.98, *P* = 0.038).

**Table 1:** Association analysis between *p53* gene and cervical cancer^a^.

Genetic Model	Genotype	Control N (%)	Cervical Cancer N (%)	OR (95% CI)	*P*	AIC	BIC
**Codominant**	G/G	29 (31.5%)	24 (43.6%)	1	0.1	157.2	211
	G/C	40 (43.5%)	22 (40.0%)	0.62 (0.23-1.69)			
	C/C	23 (25.0%)	9 (16.4%)	**0.24 (0.06-0.95)**			
**Dominant**	G/G	29 (31.5%)	24 (43.6%)	1	0.11	157.2	208
	G/C-C/C	63 (68.5%)	31 (56.4%)	0.48 (0.19-1.20)			
**Recessive**	G/G-G/C	69 (75.0%)	46 (83.6%)	1	0.055	156.1	206.9
	C/C	23 (25.0%)	9 (16.4%)	0.31 (0.09-1.09)			
**Overdominant**	G/G-C/C	52 (56.5%)	33 (60.0%)	1	0.92	159.7	210.6
	G/C	40 (43.5%)	22 (40.0%)	0.95 (0.39-2.34)			
**Log-additive**	—	—	—	**0.52 (0.27-0.98)**	**0.038**	**155.4**	**206.3**

a Adjusted for age and HPV variables.

The bold values are used to highlight data points that are central to the study’s key findings or critical for interpreting the results, as their significance is explicitly discussed in the main text.

### 3.3 Interaction analysis of *p53* gene variants with HPV16 and HPV18 infections on cervical cancer risk

This study examined the interaction between the *p53* gene and the two most prevalent high-risk HPV types, HPV16 and HPV18. **Supplementary Table S4** presents the interaction analysis between *p53* genotypes and HPV16 status, with data adjusted for age and other HPV types (including HPV18, HPV31, HPV33, HPV39, HPV45, HPV51, HPV52, HPV56, HPV58, HPV59, HPV66, HPV68, and HPV82). Individuals with the G/G genotype and HPV16 infection exhibited a significantly increased risk of cervical cancer, with an odds ratio (OR) of 16.63. This finding suggests that HPV16 infection exerts a strong carcinogenic effect in individuals with the G/G genotype. For those with the G/C or C/C genotypes, the risk of cervical cancer increased 6.38 times when infected with HPV16. While HPV16 infection increases cervical cancer risk in both genotypes, the magnitude of risk is lower for individuals with the G/C or C/C genotype compared to those with the G/G genotype. Despite this difference, the interaction *P*-value was 0.84, indicating no statistically significant interaction between *p53* genotypes and HPV16 infection, and suggesting that the two factors independently influence cervical cancer risk.

In contrast, as shown in **Supplementary Table S5**, the interaction between HPV18 infection and the *p53* genotype on cervical cancer risk was statistically significant (*P* = 0.026). This finding indicates that the *p53* genotype modulates cervical cancer risk in individuals infected with HPV18. **Table 2** demonstrates that, among HPV18-negative individuals, those with the G/C or C/C genotype had a 31% lower risk of cervical cancer compared to the G/G genotype, although this difference did not reach statistical significance. However, among HPV18-positive individuals, using the G/G genotype as the reference (OR = 1), the OR for cervical cancer risk in individuals with the G/C or C/C genotype was 0. This suggests that the *p53* genotype may influence cervical cancer risk in the context of HPV18 infection. Specifically, individuals carrying the C allele may have some protective effect against cervical cancer in the presence of HPV18, while those with the G/G genotype may experience a significantly increased risk following HPV18 infection.

**Table 2:** Impact of different *p53* genotypes on cervical cancer risk in HPV18 infection^a^.

HPV18	Genotype	Control	Cervical Cancer	OR (95% CI)	*P*
**Negative**	G/G	29	17	1	**0.026**
	G/C-C/C	57	27	0.69 (0.26-1.85)	
**Positive**	G/G	0	7	1	
	G/C-C/C	6	4	**0**	

a Adjusted for age and other HPV types including HPV16, HPV31, HPV33, HPV39, HPV45, HPV51, HPV52, HPV56, HPV58, HPV59, HPV66, HPV68, HPV82.

The bold values are used to highlight data points that are central to the study’s key findings or critical for interpreting the results, as their significance is explicitly discussed in the main text.

### 3.4 Hematological parameters and inflammatory biomarkers in cervical cancer patients

As presented in **Table 3**, significant differences were observed between the cervical cancer group and the control group across several hematological parameters, particularly in RBC, HGB, NEUT%, LY, MONO, and MONO%. These findings indicate that cervical cancer patients may exhibit distinct hematological alterations and inflammatory responses. Such results provide valuable insights for the early diagnosis and clinical monitoring of cervical cancer. In particular, potential clinical biomarkers, such as the NLR, SII and PLR, may hold significant promise in the evaluation and assessment of cervical cancer.

**Table 3:** Comparing blood counts and inflammatory markers in two groups.

Variable	Control (n = 92)	Cervical Cancer (n = 55)	*P*
WBC, Mean ± SD	6.4 ± 1.23	6.07 ± 1.3	0.131
RBC, Mean ± SD	4.53 ± 0.39	4.37 ± 0.39	**0.025**
HGB, Median (Q1,Q3)	134.5 (127, 142)	130 (117.5, 137.5)	**0.01**
PLT, Mean ± SD	236.92 ± 54.83	243.38 ± 73.59	0.574
NEUT, Mean ± SD	3.87 ± 1.04	4.03 ± 1.09	0.375
NEUT%, Mean ± SD	59.96 ± 8.53	65.85 ± 6.71	**< 0.001**
EO, Median (Q1,Q3)	0.07 (0.04, 0.12)	0.06 (0.03, 0.1)	0.112
EO%, Median (Q1,Q3)	1.2 (0.7, 2.1)	1.1 (0.55, 1.65)	0.222
BASO, Median (Q1,Q3)	0.03 (0.02, 0.04)	0.03 (0.02, 0.03)	0.502
BASO%, Median (Q1,Q3)	0.4 (0.3, 0.6)	0.5 (0.3, 0.6)	0.96
LY, Median (Q1,Q3)	1.95 (1.55, 2.32)	1.62 (1.38, 1.83)	**< 0.001**
LY%, Mean ± SD	31.09 ± 7.55	27.16 ± 6.24	**< 0.001**
MONO, Median (Q1,Q3)	0.41 (0.34, 0.49)	0.29 (0.24, 0.39)	**< 0.001**
MONO%, Median (Q1,Q3)	6.4 (5.55, 7.6)	5.1 (4.1, 6.3)	**< 0.001**
HCT, Median (Q1,Q3)	0.4 (0.38, 0.42)	0.39 (0.35, 0.42)	0.106
RDWCV, Median (Q1,Q3)	12.5 (12, 13.12)	12.5 (12.1, 13.1)	0.646
MCH, Median (Q1,Q3)	30.1 (29.17, 31)	29.8 (28.6, 30.6)	0.123
MCHC, Median (Q1,Q3)	335 (331, 341.25)	329 (321.5, 335)	**< 0.001**
SIRI, Median (Q1,Q3)	0.75 (0.64, 1.11)	0.76 (0.5, 1.06)	0.381
NLR, Median (Q1,Q3)	1.92 (1.54, 2.54)	2.51 (1.9, 3)	**< 0.001**
SII, Median (Q1,Q3)	453.22 (328.36, 638.1)	548.09 (417.41, 790.42)	**0.003**
PLR, Median (Q1,Q3)	118.87 (101.92, 142.66)	145.71 (124.22, 178.7)	**< 0.001**
LMR, Median (Q1,Q3)	4.84 (3.88, 5.68)	5.23 (3.93, 6.86)	0.073
NPR, Median (Q1,Q3)	0.02 (0.01, 0.02)	0.02 (0.01, 0.02)	0.747

The bold values are used to highlight data points that are central to the study’s key findings or critical for interpreting the results, as their significance is explicitly discussed in the main text.

### 3.5 Logistic regression and LASSO analysis for cervical cancer risk prediction

A univariate logistic regression analysis was conducted on variables including high-risk HPV, *p53* genotypes (G/G and G/C-C/C), blood routine parameters, and inflammatory markers. Using a significance threshold of *P <* 0.20, a total of 23 variables were selected for further analysis (**Supplementary Table S6**). Subsequently, a LASSO regression analysis with 10-fold cross-validation was applied to these 23 variables (**Supplementary Figure S1**), and the optimal regularization parameter λ was identified (lambda.1SE = 0.05218946). Eight key variables were found to be significantly associated with cervical cancer risk: *p53* genotype, HPV16, HPV18, MONO, MONO%, NEUT%, MCH, and RBC. These variables exhibited strong predictive value for cervical cancer within the model, laying a solid foundation for subsequent multivariate logistic regression analysis and clinical applications.

### 3.6 Development and validation of a nomogram prediction model for cervical cancer risk

**Figure 1:**
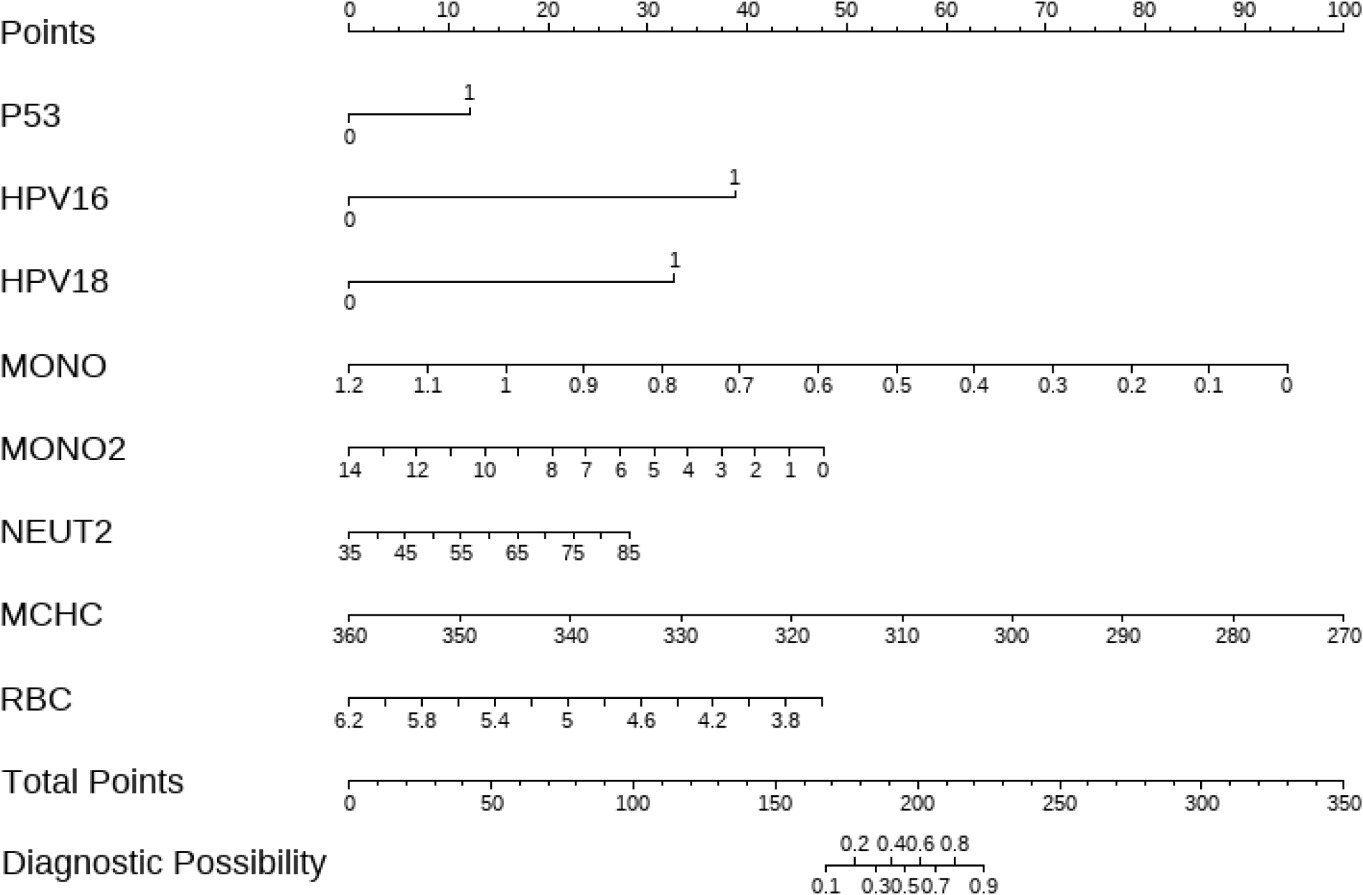
Clinical nomogram for predicting cervical cancer diagnosis. *p53*: 0 represents GC or CC genotype, 1 represents GG genotype. HPV16 and HPV18: 0 represents negative, 1 represents positive.

A multivariate logistic regression analysis was performed using the 8 key variables identified through LASSO regression. BACKWARD was applied to select the final variables, as presented in **Table 4**. Based on these selected variables, a nomogram prediction model was constructed (**Figure 1**). This model calculates an individual score for each predictor and generates a total score to estimate the probability of cervical cancer occurrence, thus assisting in the diagnosis of cervical cancer.

**Table 4:** Multivariate logistic regression analysis of selected variables.

No.	Variable	B	SE	OR (95% CI)	Z	*P*
1	*p53*	0.959	0.57458	2.609 (0.860-8.403)	1.669	0.095
2	HPV16	3.066	0.71488	21.45 (5.815-99.48)	4.289	0
3	HPV18	2.587	0.76848	13.29 (3.139-66.28)	3.367	0.001
4	MONO	-6.219	4.04638	0.001 (5.914-5.433)	-1.537	0.124
5	MONO%	-0.269	0.31192	0.763 (0.396-1.359)	-0.863	0.388
6	NEUT%	0.045	0.03707	1.045 (0.973-1.127)	1.202	0.229
7	MCHC	-0.088	0.02466	0.915 (0.868-0.957)	-3.566	0
8	RBC	-1.448	0.71577	0.235 (0.052-0.890)	-2.023	0.043

**Figure 2:**
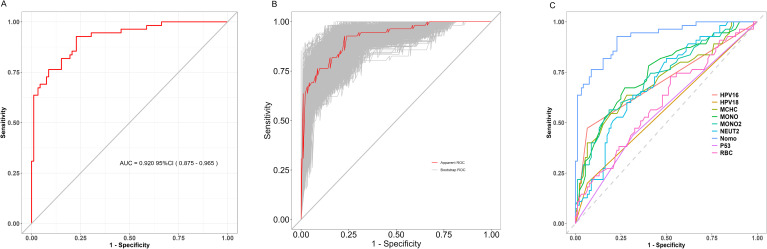
**(A)** ROC curve; **(B)** Bootstrap Validation of Model Stability (1000 Resampling Iterations); **(C)** ROC Curve Analysis of Diagnostic Model Performance.

The model’s discriminative ability was evaluated using a ROC curve (**Figure 2A**) and validated with 1000 bootstrap resampling iterations (**Figure 2B**). The results demonstrated an AUC of 0.920 (95% CI: 0.875–0.965), indicating strong performance in distinguishing cervical cancer cases from non-cancer cases. Moreover, the ROC rationality analysis (**Figure 2C**) showed that the nomogram model’s curve was the closest to the upper left corner, further confirming its superior performance compared to individual variable models in differentiating between cervical cancer and non-cervical cancer cases.

**Figure 3:**
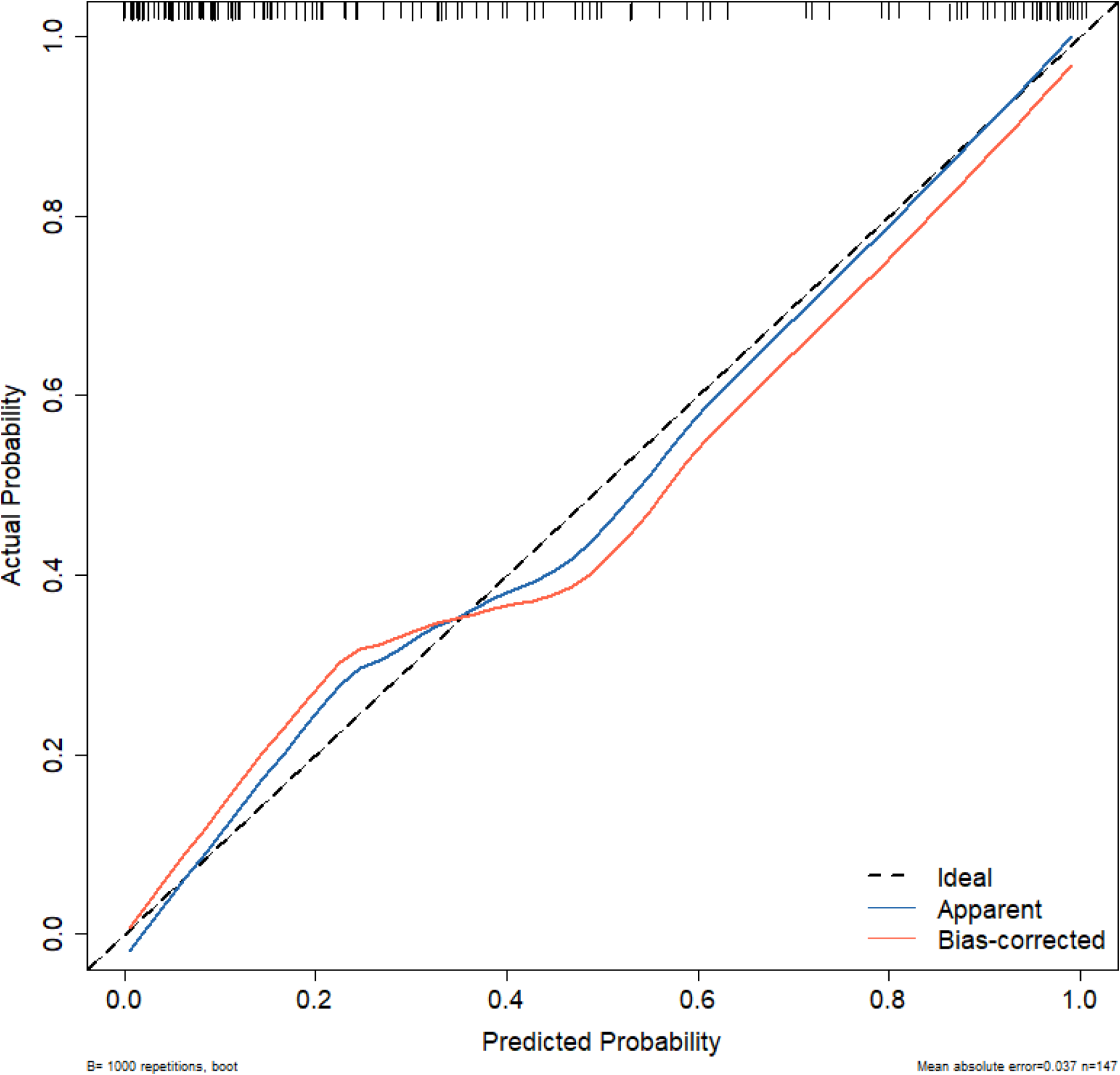
Calibration curve for the nomogram model. Ideal: The ideal curve; Apparent: The original data model; Bias-corrected: The model prediction after 1000 bootstrap resampling corrections.

The calibration of the model was further assessed using the Hosmer-Lemeshow goodness-of-fit test and a calibration curve. The Hosmer-Lemeshow test yielded a *χ²* value of 5.1654, *P =* 0.7398 (*P >* 0.05), indicating no statistically significant difference between the predicted and observed values. **Figure 3** shows the proximity between the prediction curve, drawn from 1000 bootstrap resamples, and the reference line, demonstrating good agreement between the model’s predictions and actual outcomes.

**Figure 4:**
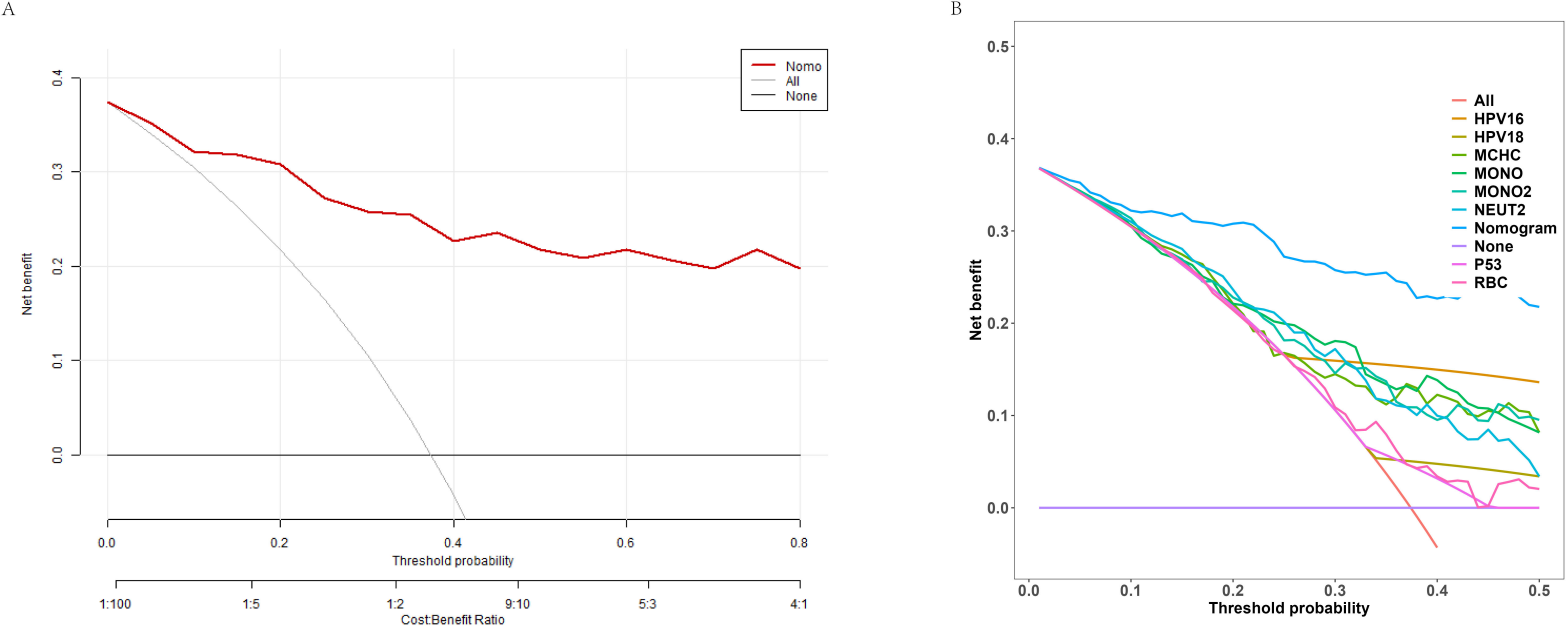
Decision curve analysis (DCA) of the nomogram model. **(A)** Net benefit curve of the nomogram predictive model at different decision probability thresholds. **(B)** Comparison of net benefits between different variables and the predictive model at various probability thresholds.

Lastly, **Figure 4** presents the DCA, generated from 1000 bootstrap resamples. The results suggest that the model provides substantial clinical benefit and can be a valuable tool for guiding clinical decision-making.

## 4 Discussion

The association analysis between the *p53* gene and cervical cancer (**Table 1**) revealed that the C/C genotype significantly reduced the risk of cervical cancer, suggesting a potential protective effect of the C allele, which aligns with findings from previous studies (8, 17). Although statistical significance was not achieved in other models, the results indicated that the C allele might be associated with a lower risk of cervical cancer. The log-additive model results further support the idea that the C allele may exert a dose-dependent protective effect. These findings suggest that the *p53* genotype may influence cervical cancer risk through a complex genetic interplay. Overall, these results provide valuable insights for further investigation into the role of the *p53* genotype in the development of cervical cancer.

Infection with HPV16 significantly increases the risk of cervical cancer, with a more pronounced risk observed in individuals with the G/G genotype. This finding may suggest that the C allele offers some degree of protection; however, no significant interaction between the two was identified. This insight enhances our understanding of the complex relationship between genotype and HPV infection in the development of cervical cancer, which should be further validated in larger-scale studies in the future.

In individuals infected with HPV18, those with the G/C or C/C genotype exhibited an OR of 0 when compared to the G/G genotype (**Table 2**). This finding is particularly intriguing, as an OR of 0 suggests that individuals carrying at least one C allele (G/C or C/C) appear to have no risk, or an extremely low risk, of developing cervical cancer in the context of HPV18 infection. The *p53* genotype significantly influences cervical cancer risk among HPV18-infected individuals, indicating that different *p53* variants may have varying effects in response to HPV18.

The G/C and C/C genotypes appear to confer a strong protective effect against cervical cancer in those infected with HPV18, suggesting that the C allele could play a crucial role in resisting cancer progression induced by HPV18, potentially through more effective suppression of HPV18-driven oncogenesis. If validated, these results could have significant implications for risk assessment and management strategies in HPV18-infected individuals, with those carrying the G allele possibly requiring tailored follow-up and intervention protocols. However, it is essential to note that the sample size of this study (n = 147) is relatively small, which may affect the stability and generalizability of the findings. Furthermore, an OR of 0 is an extreme outcome, highlighting the need for larger studies to validate this result and explore the underlying mechanisms further.

Significant differences were observed in several blood routine parameters between the cervical cancer group and the control group. The reduction in RBC and HGB may indicate an anemic state in cervical cancer patients, potentially linked to the biological characteristics of the tumor, malnutrition, or chronic bleeding. The NEUT% and decrease in LY suggest the presence of a systemic inflammatory response, which may be associated with the tumor’s immune evasion mechanisms (18). Additionally, the decrease in MONO and its percentage may reflect immune suppression (19, 20). It is noteworthy that age may also influence hematologic parameters (21). For instance, increasing patient age may lead to decreased hemoglobin levels and affect systemic inflammatory indices. In this study, we implemented an age-matched design between the two groups to mitigate such confounding effects. These findings provide critical insights for the early diagnosis and clinical monitoring of cervical cancer. Specifically, indicators such as the NLR, SII, and PLR may possess significant clinical value in assessing cervical cancer. This underscores the rationale for including these parameters in the predictive model for cervical cancer developed in this study.

Following the univariate logistic regression and LASSO regression analyses of variables such as high-risk HPV, *p53* genotypes, and blood routine indicators, eight variables were identified as being significantly associated with cervical cancer risk: *p53* genotype, HPV16, HPV18, MONO, MONO%, NEUT%, MCH, and RBC. The significance of HPV16 and HPV18 underscores their critical roles in the development of cervical cancer, aligning with findings in the existing literature (22). Furthermore, the significance of MONO and MONO% indicates that variations in monocyte counts may be linked to immune responses within the tumor microenvironment (23). Additionally, the significance of MCH and RBC suggests that red blood cells and related parameters may also play an important role in the pathogenesis of cervical cancer.

The AUC was 0.920, demonstrating that the model developed in this study has a remarkably high discriminatory ability, effectively distinguishing cervical cancer patients from non-cancer cases. Furthermore, the results of the Hosmer-Lemeshow goodness-of-fit test and calibration curve (*χ²* = 5.1654, *P =* 0.7398) indicate that the model’s predictions closely align with actual outcomes. The DCA further shows that in the moderate to high probability threshold range, the model provides a substantial net benefit, reinforcing its practical value in clinical applications.

In conclusion, this study analyzed the *p53* genotype, high-risk HPV types, and routine blood parameters in patients with cervical cancer and a control group. We identified significant differences between the two groups and highlighted the potential clinical value of these variables. Key variables associated with cervical cancer risk were identified, and an effective predictive model was constructed. This model demonstrated good discrimination and calibration, and showed substantial benefits in clinical decision-making, providing new insights for preliminary screening and risk prediction of cervical cancer. The study also emphasized the interaction between HPV infection and *p53* tumor suppressor gene mutations, highlighting their importance in cancer development. In particular, the interaction between HPV16, HPV18, and *p53* genotypes, and its impact on cervical cancer onset, was explored—a topic that has not been thoroughly addressed in existing literature. Future studies should further validate the applicability of this model in larger sample sizes and investigate its potential use in other cancer types. By optimizing detection methods and risk assessment tools, we hope to provide more effective strategies for clinical practice to reduce the incidence and mortality of cervical cancer.

## Data Availability

The original contributions presented in the study are included in the article/**Supplementary Material**. Further inquiries can be directed to the corresponding authors.
