# Supplementary figures and images for: Innovative nomogram for cervical cancer prediction: integrating high-risk HPV infection, p53 genotype, and blood routine parameters
Source: Front Oncol. 2025 May 20;15:1541928. doi: 10.3389/fonc.2025.1541928 (PMC12129748; doi:10.3389/fonc.2025.1541928)

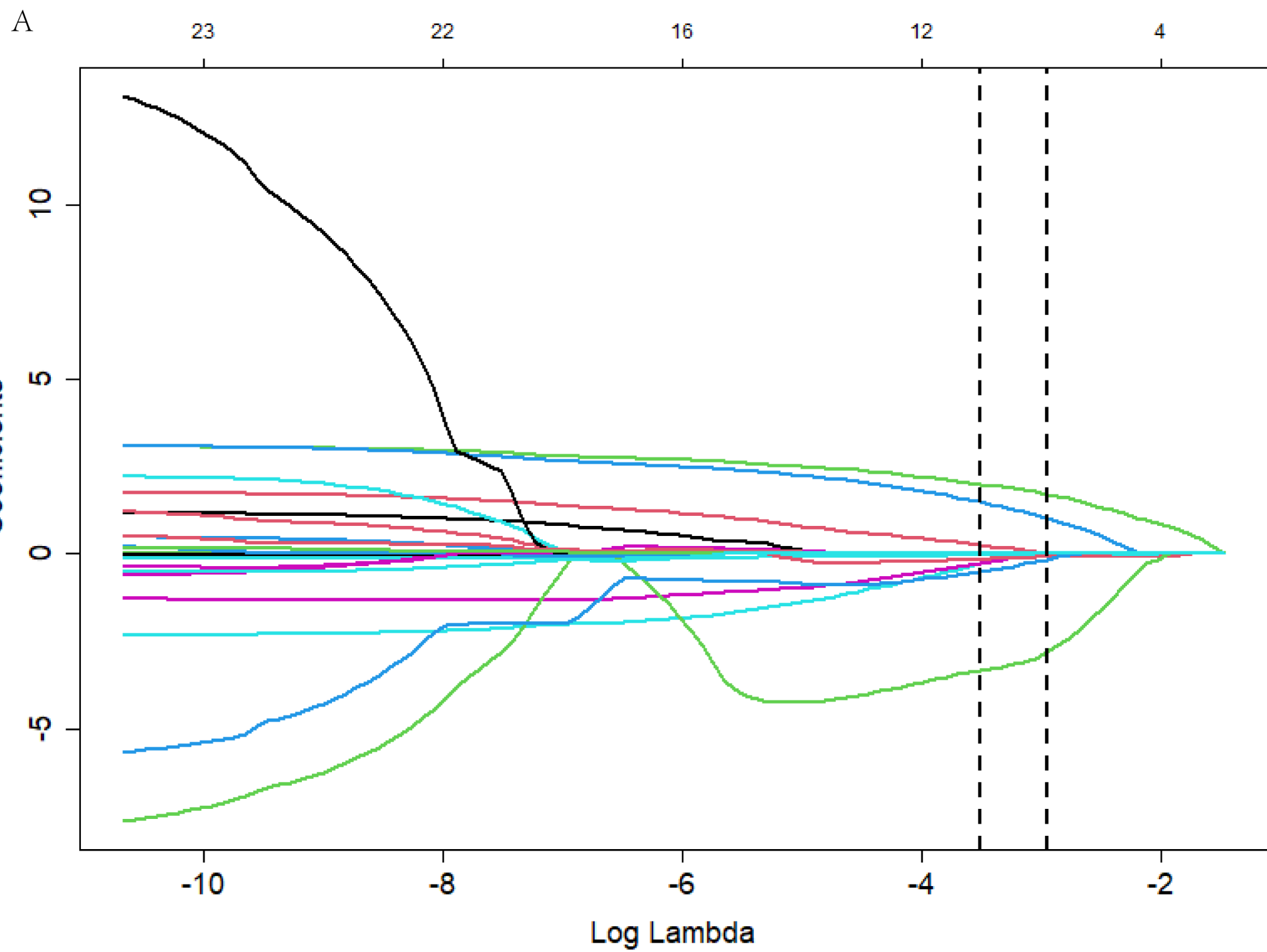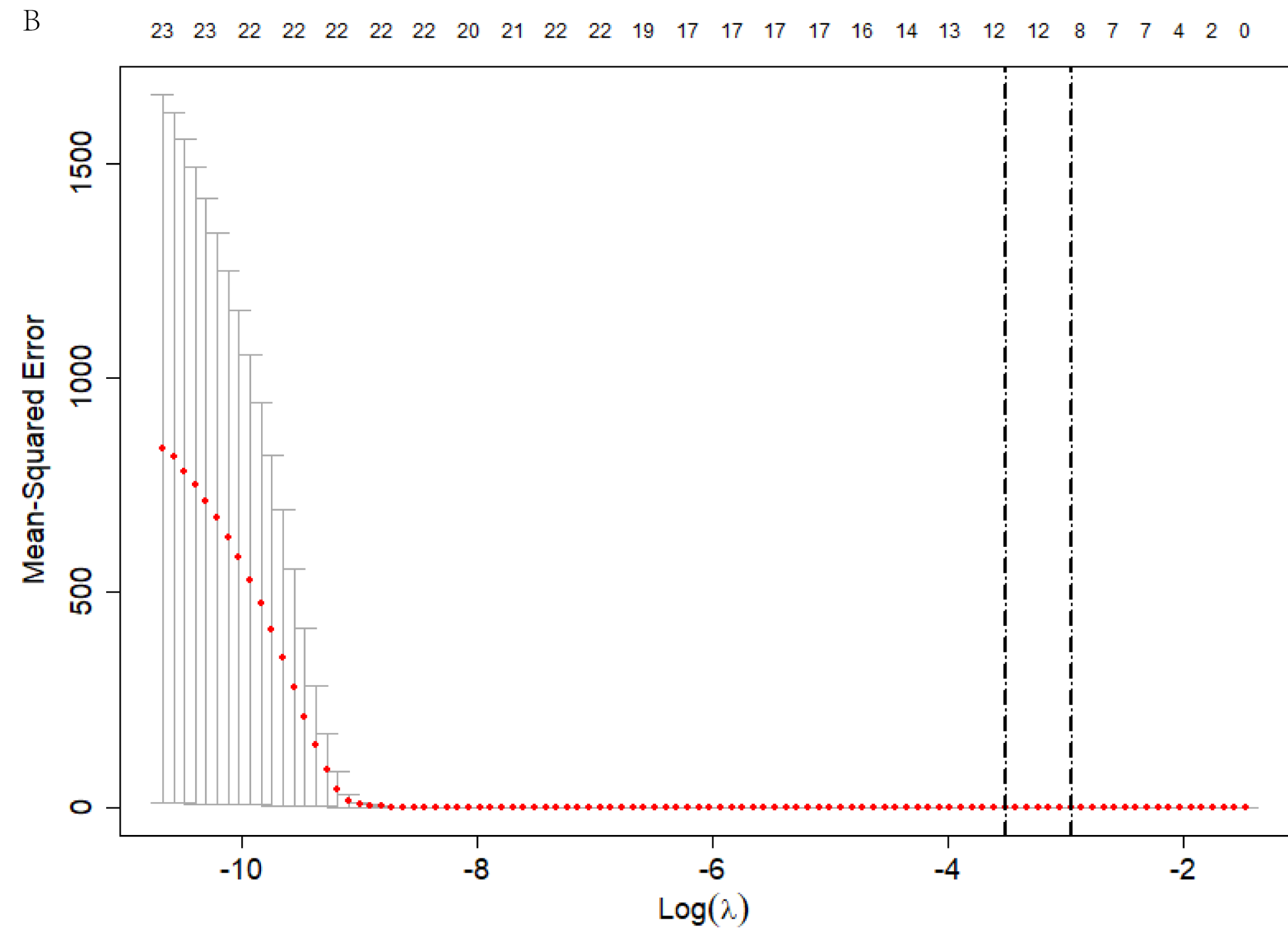

LASSO regression analysis plot. (A) LASSO path diagram. (B) LASSO 10-fold cross-validation plot.

Supplement: Supplementary file 1 [file DataSheet1.pdf]
