# Supplementary material for: Innovative nomogram for cervical cancer prediction: integrating high-risk HPV infection, p53 genotype, and blood routine parameters
Source: Front Oncol. 2025 May 20;15:1541928. doi: 10.3389/fonc.2025.1541928 (PMC12129748; doi:10.3389/fonc.2025.1541928)
Supplement: Supplementary file 2 [file Table1.docx]

**Table S1.** Comparing age and high-risk HPV rates in two groups

| **Variable** | **Total (n = 147)** | **Control Group (n = 92)** | **Cervical Cancer Group (n = 55)** | ***P*** |
| --- | --- | --- | --- | --- |
| Age, Mean ± SD | 48.76 ± 11.26 | 47.2 ± 11.75 | 51.36 ± 9.93 | 0.023 |
| HPV16, n (%) |  |  |  | **< 0.001** |
| Negative | 115 (78) | 86 (93) | 29 (53) |  |
| Positive | 32 (22) | 6 (7) | 26 (47) |  |
| HPV18, n (%) |  |  |  | **0.027** |
| Negative | 130 (88) | 86 (93) | 44 (80) |  |
| Positive | 17 (12) | 6 (7) | 11 (20) |  |
| HPV31, n (%) |  |  |  | 1 |
| Negative | 141 (96) | 88 (96) | 53 (96) |  |
| Positive | 6 (4) | 4 (4) | 2 (4) |  |
| HPV33, n (%) |  |  |  | 0.374 |
| Negative | 146 (99) | 92 (100) | 54 (98) |  |
| Positive | 1 (1) | 0 (0) | 1 (2) |  |
| HPV39, n (%) |  |  |  | 0.084 |
| Negative | 141 (96) | 86 (93) | 55 (100) |  |
| Positive | 6 (4) | 6 (7) | 0 (0) |  |
| HPV45, n (%) |  |  |  | 0.374 |
| Negative | 146 (99) | 92 (100) | 54 (98) |  |
| Positive | 1 (1) | 0 (0) | 1 (2) |  |
| HPV51, n (%) |  |  |  | 0.157 |
| Negative | 142 (97) | 87 (95) | 55 (100) |  |
| Positive | 5 (3) | 5 (5) | 0 (0) |  |
| HPV52, n (%) |  |  |  | 0.154 |
| Negative | 138 (94) | 84 (91) | 54 (98) |  |
| Positive | 9 (6) | 8 (9) | 1 (2) |  |
| HPV56, n (%) |  |  |  | 0.157 |
| Negative | 142 (97) | 87 (95) | 55 (100) |  |
| Positive | 5 (3) | 5 (5) | 0 (0) |  |
| HPV58, n (%) |  |  |  | **0.032** |
| Negative | 134 (91) | 80 (87) | 54 (98) |  |
| Positive | 13 (9) | 12 (13) | 1 (2) |  |
| HPV59, n (%) |  |  |  | 1 |
| Negative | 144 (98) | 90 (98) | 54 (98) |  |
| Positive | 3 (2) | 2 (2) | 1 (2) |  |
| HPV66, n (%) |  |  |  | 0.528 |
| Negative | 145 (99) | 90 (98) | 55 (100) |  |
| Positive | 2 (1) | 2 (2) | 0 (0) |  |
| HPV68, n (%) |  |  |  | 0.528 |
| Negative | 145 (99) | 90 (98) | 55 (100) |  |
| Positive | 2 (1) | 2 (2) | 0 (0) |  |
| HPV82, n (%) |  |  |  | 1 |
| Negative | 146 (99) | 91 (99) | 55 (100) |  |
| Positive | 1 (1) | 1 (1) | 0 (0) |  |

**Table S2.** Allele and genotype frequencies of *p53* gene in the two groups

|  | **Control Group** | |  | **Cervical Cancer Group** | |
| --- | --- | --- | --- | --- | --- |
|  | **Count** | **Frequency** |  | **Count** | **Frequency** |
| **Alleles** |  |  |  |  |  |
| G | 98 | 0.53 |  | 70 | 0.64 |
| C | 86 | 0.47 |  | 40 | 0.36 |
| **Genotypes** |  |  |  |  |  |
| C/C | 23 | 0.25 |  | 9 | 0.16 |
| G/C | 40 | 0.43 |  | 22 | 0.4 |
| G/G | 29 | 0.32 |  | 24 | 0.44 |

**Table S3.** Hardy-Weinberg Equilibrium test for *p53* gene

|  | **GG** | **GC** | **CC** | **G** | **C** | ***P*** |
| --- | --- | --- | --- | --- | --- | --- |
| **All patients (n)** | 53 | 62 | 32 | 168 | 126 | 0.094 |
| **Control group (n)** | 29 | 40 | 23 | 98 | 86 | 0.22 |
| **Cervical cancer group (n)** | 24 | 22 | 9 | 70 | 40 | 0.38 |

**Table S4.** Interaction between *p53* genotypes and HPV16^a^

|  | **HPV16 Negative** | | |  | **HPV16 Positive** | | | ***P*** |
| --- | --- | --- | --- | --- | --- | --- | --- | --- |
|  | **Control** | **Cervical Cancer** | **OR**  **(95% CI)** |  | **Control Group** | **Cervical Cancer** | **OR**  **(95% CI)** |  |
| **G/G** | 28 | 15 | 1 |  | 1 | 9 | **16.63**  (1.79-154.89) | 0.84 |
| **G/C-C/C** | 58 | 14 | 0.50  (0.18-1.37) |  | 5 | 17 | **6.38**  (1.78-22.93) |  |

^a^Adjusted for age and other HPV types including HPV18, HPV31, HPV33, HPV39, HPV45, HPV51, HPV52, HPV56, HPV58, HPV59, HPV66, HPV68, HPV82

**Table S5.** Interaction Between *p53* Genotypes and HPV18^a^

| **Genotype** | **HPV18 Negative** | | |  | **HPV18 Positive** | | | ***P*** |
| --- | --- | --- | --- | --- | --- | --- | --- | --- |
|  | **Control** | **Cervical Cancer** | **OR**  **(95% CI)** |  | **Control Group** | **Cervical Cancer** | **OR**  **(95% CI)** |  |
| **G/G** | 29 | 17 | 1 |  | 0 | 7 | --- | **0.026** |
| **G/C-C/C** | 57 | 27 | 0.69  (0.26-1.85) |  | 6 | 4 | 2.35  (0.49-11.38) |  |

^a^Adjusted for age and other HPV types including HPV16, HPV31, HPV33, HPV39, HPV45, HPV51, HPV52, HPV56, HPV58, HPV59, HPV66, HPV68, HPV82

**Table S6.** Univariate logistic regression analysis of selected variables

| **No.** | **Variable** | **B** | **SE** | **OR (95% CI)** | **Z** | ***P*** |
| --- | --- | --- | --- | --- | --- | --- |
| 1 | LMR | 0.186 | 0.09924 | 1.205(1.021-1.473) | 1.878 | 0.06 |
| 2 | PLR | 0.012 | 0.00372 | 1.012(1.005-1.02) | 3.161 | 0.002 |
| 3 | SII | 0.002 | 0.0007 | 1.002(1.001-1.003) | 2.823 | 0.005 |
| 4 | NLR | 0.62 | 0.20093 | 1.86(1.274-2.815) | 3.087 | 0.002 |
| 5 | MCHC | -0.064 | 0.0172 | 0.938(0.904-0.967) | -3.726 | 0 |
| 6 | MCH | -0.101 | 0.05752 | 0.903(0.803-1.01) | -1.764 | 0.078 |
| 7 | HCT | -9.431 | 4.83004 | 0(0-0.151) | -1.953 | 0.051 |
| 8 | MONO% | -0.591 | 0.13559 | 0.554(0.417-0.71) | -4.361 | 0 |
| 9 | MONO | -8.268 | 1.85205 | 0(0-0.008) | -4.464 | 0 |
| 10 | LY% | -0.081 | 0.02652 | 0.922(0.873-0.969) | -3.059 | 0.002 |
| 11 | LY | -1.619 | 0.42129 | 0.198(0.083-0.435) | -3.843 | 0 |
| 12 | EO% | -0.182 | 0.1306 | 0.833(0.618-1.033) | -1.396 | 0.163 |
| 13 | EO | -2.926 | 2.03361 | 0.054(0-1.503) | -1.439 | 0.15 |
| 14 | NEUT% | 0.099 | 0.02528 | 1.104(1.053-1.163) | 3.904 | 0 |
| 15 | HGB | -0.038 | 0.01266 | 0.963(0.938-0.986) | -2.975 | 0.003 |
| 16 | RBC | -1.026 | 0.46566 | 0.358(0.139-0.867) | -2.204 | 0.027 |
| 17 | WBC | -0.213 | 0.13934 | 0.808(0.611-1.058) | -1.527 | 0.127 |
| 18 | HPV58 | -2.092 | 1.05563 | 0.123(0.007-0.654) | -1.982 | 0.048 |
| 19 | HPV52 | -1.638 | 1.07488 | 0.194(0.01-1.103) | -1.524 | 0.128 |
| 20 | HPV18 | 1.276 | 0.54031 | 3.583(1.277-11.00) | 2.362 | 0.018 |
| 21 | HPV16 | 2.553 | 0.50124 | 12.85(5.097-37.38) | 5.094 | 0 |
| 22 | *p53* | 0.52 | 0.35253 | 1.682(0.842-3.368) | 1.475 | 0.14 |
| 23 | Age | 0.034 | 0.016 | 1.035(1.004-1.069) | 2.147 | 0.032 |
